# Methylation of *p15^INK4b^* and Expression of *ANRIL* on Chromosome 9p21 Are Associated with Coronary Artery Disease

**DOI:** 10.1371/journal.pone.0047193

**Published:** 2012-10-16

**Authors:** Jianhui Zhuang, Wenhui Peng, Hailing Li, Wei Wang, Yidong Wei, Weiming Li, Yawei Xu

**Affiliations:** 1 Department of Cardiology, Shanghai Tenth People's Hospital, Tongji University School of Medicine, Shanghai, China; 2 Laboratory of Blood and Vascular Biology, The Rockefeller University, New York, New York, United States of America; Leibniz-Institute for Arteriosclerosis Research at the University Muenster, Germany

## Abstract

**Background:**

Genome-wide association studies have identified that multiple single nucleiotide polymorphisms on chromosome 9p21 are tightly associated with coronary artery disease (CAD). However, the mechanism linking this risk locus to CAD remains unclear.

**Methodology/Principal Findings:**

The methylation status of six candidate genes (*BAX*, *BCL-2*, *TIMP3*, *p14^ARF^*, *p15^INK4b^* and *p16^INK4a^*) in 205 patients and controls who underwent coronary angiography were analyzed by quantitative MethyLight assay. Rs10757274 was genotyped and expression of *INK4/ARF* and antisense non-coding RNA in the INK4 locus (*ANRIL*) was determined by real-time RT-PCR. Compared with controls, DNA methylation levels at *p15^INK4b^* significantly increased in CAD patients (p = 0.006). To validate and dissect the methylation percentage of each target CpG site at *p15^INK4b^*, pyrosequencing was performed, finding CpG +314 and +332 remarkably hypermethylated in CAD patients. Further investigation determined that *p15^INK4b^* hypermethylation prevalently emerged in lymphocytes of CAD patients (p = 0.013). The rs10757274 genotype was significantly associated with CAD (p = 0.003) and GG genotype carriers had a higher level of *ANRIL exon 1–5* expression compared among three genotypes (p = 0.009). There was a stepwise increase in *p15^INK4b^* and *p16^INK4a^* methylation as *ANRIL exon 1–5* expression elevated (r = 0.23, p = 0.001 and r = 0.24, p = 0.001, respectively), although neither of two loci methylation was directly linked to rs10757274 genotype.

**Conclusions/Significance:**

*p15^INK4b^* methylation is associated with CAD and *ANRIL* expression. The epigenetic changes in *p15^INK4b^* methylation and *ANRIL* expression may involve in the mechanisms of chromosome 9p21 on CAD development.

## Introduction

**Figure 1:**
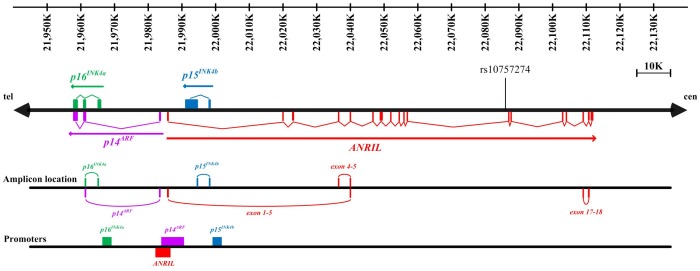
Overview of human locus on chromosome 9p21. The exons and promoters of *p14^ARF^*, *p15^INK4b^* and *p16^INK4a^* are shown in purple, blue and green respectively (intron structures not shown). The exons and promoter of *ANRIL* transcribed in opposite direction are shown in red. The amplicon locations of *p14^ARF^*, *p15^INK4b^*, *p16^INK4a^* and *ANRIL* transcripts are indicated in the same color as filled in their exons. Abbreviations: *ANRIL* = antisense non-coding RNA in the INK4 locus, cen = centromere, tel = telomere.

Genome-wide association studies (GWAS) have found that single nucleotide polymorphisms (SNPs) on chromosome 9p21 (Chr9p21) affect susceptibility to coronary artery disease (CAD) in Caucasian population [1], [2], [3], [4], and these associations have been reproduced in other populations [5], [6], [7], [8]. However, the mechanisms of Chr9p21 for CAD remain elusive. Most of SNPs are highly correlated and located within a roughly 53-kb linkage disequilibrium (LD) region in which a long non-coding RNA, known as antisense non-coding RNA in the INK4 locus (*ANRIL*), is transcribed. It has been extensively reported that *ANRIL* transcripts are assembled with many exons and multiple isoforms of *ANRIL* transcripts coexist in a variety of cell types [9]. The genetic sequences upstream to Chr9p21 encode a well-characterized cluster of tumor suppressor genes, *p14^ARF^*, *p15^INK4b^* and *p16^INK4a^*, alias *INK4/ARF*, all of which are transcribed from the opposite strand to *ANRIL* (Figure 1).

Previous studies show that both deletion of Chr9p21 locus and repression of *INK4/ARF* or *ANRIL* expression have their impacts on atherosclerosis [10], [11]. Further studies find that multiple SNPs in the risk haplotype region may have substantial influences on *ANRIL* expression levels or *ANRIL* splices, while *ANRIL* epigenetically regulates *INK4/ARF* expression [12], [13], [14], [15], [16], [17], [18], [19], [20].

Epigenetics is defined as stable and heritable changes that are not due to disrupting the coding sequences of disease genes which has been shown to play an important role in various diseases including cancer, type 2 diabetes, systemic lupus erythematosus, etc. [21], [22], [23]. Cancer cells armed with both a loss of global methylation and a gain of methylation at tumor suppressor genes, such as *INK4/ARF*, often show unlimited proliferation [24], [25]. Atherosclerostic plaque also has characteristics of excessive vascular smooth muscle cells (VSMCs) and macrophages proliferation. Inspired by these evidences and the strong association of Chr9p21 with CAD, we hypothesized that *INK4/ARF* hypermethylation also had its role in CAD development. Due to the little knowledge about the role of DNA methylation of specific loci in chromosome on cardiovascular disease so far, this study would help to explore a novel aspect in understanding cardiovascular disease.

In this study, we sought to determine whether DNA methylations of selected CpG islands, especially those in *INK4/ARF* locus, were involved in CAD. Since multiple SNPs associated with CAD do not appear to directly affect *INK4/ARF* expression, the altered expression of *INK4/ARF* is likely to be modulated by *ANRIL* or other epigenetic changes. Toward this end, we attempted to explore the association of DNA methylation with risk genotypes and altered *ANRIL* expression on Chr9p21.

## Methods

### Ethnic statement

This study was approved by the Ethics Committee of Shanghai Tenth People's Hospital. All patients gave written informed consent.

### Study population

A total of 95 patients who were diagnosed as CAD by angiography and 110 sex- and age-matched participants without CAD after angiography were recruited in Department of Cardiology, Shanghai Tenth People's Hospital from March 2011 to October 2011. Of note, patients with cancer, acute myocardial infarction, severe heart failure (left ventricular ejection fraction ≤30%), cardiomyopathy, active infection and connective tissue disease were excluded. Hypertension was defined as systolic or diastolic blood pressure ≥140/90 mm Hg, under anti-hypertensive medications for one year before admission. Diabetes was defined as fasting blood glucose ≥7 mmol/L, non-fasting plasma glucose level ≥11.1 mmol/L or known treatment for diabetes. Peripheral venous blood (20 mL) was drawn into adequate tubes from each subject. A white differential cell count on whole blood using automated counter was performed.

### Coronary angiography

Quantitative assessment of CAD was performed using coronary angiography as previously described [26]. In brief, significant CAD was defined as the presence of luminal diameter narrowing ≥50% in the left anterior descending artery, left circumflex artery, right coronary artery and their main branches. Left main trunk stenosis was considered as two-vessel disease. Severity of coronary atherosclerosis was further categorized as 1-, 2- or ≥3-vessel disease according to number of coronary vessels with significant stenosis.

### Isolation of neutrophils and lymphocytes from peripheral blood

Human neutrophils and lymphocytes were isolated from 5 ml heparin-anticoagulated blood drawn from 26 healthy participants and 38 CAD patients as previously described [27], with minor modifications. Briefly, the 1∶1 mixtures of peripheral blood and HBSS (without Ca^2+^ and Mg^2+^) were added into 2 ml Ficoll-Paque Plus (GE Healthcare, USA), followed by centrifugation at 1,500 rpm for 15 min without brake. Lymphocytes were collected at the interphase and neutrophils were collected by carefully removing the layer immediately above the red blood cells, followed by addition of 6% Dextran 500 prepared in 0.9% NaCl solution. After allowing RBC to settle for 30 to 60 min at room temperature, neutrophils in the supernatant were harvested. The cell type and purify were appraised by fluorescence-activated cell sorting as CD45-high SSC-low for lymphocytes and CD45-low SSC-high for neutrophils.

### DNA extraction and genotyping

All peripheral blood samples were taken in the morning with patients fasting from midnight onward. Genomic DNA was extracted from whole blood cells, neutrophilis and lymphocytes using commercial available kit (Tiagen Biotech, Beijing, China). Based on the validation of GWAS and independent studies [2], [12], we selected SNP rs10757274 for genotying, which is a representative marker of atherosclerostic diseases as Chr9p21 was a highly LD region. Genotyping was performed with TaqMan allelic discrimination by means of an ABI 7900HT (Applied Biosystems, CA, USA), in 384-well format. The TaqMan Assay kits as well as probes were purchased from Applied Biosystems. Data were analyzed using the ABI Prism SDS software version 2.3.

### Bisulfite treatment and MethyLight analysis

Genomic DNA was then chemically modified by sodium bisulfite to convert all unmethylated cytosines to uracils while leaving methylcytosines unaltered (EZ Zymo Methylation Kit, Zymo Research, CA, USA). DNA methylation analysis was performed by MethyLight as previously described [28]. The gene names, locations, primer and probe sequences are summarized in Table S1. The *β-Actin* (*ATCB*) repeats were used as an internal reference to normalize the input DNA and to generate a standard curve. The amount of methylated DNA was determined by the threshold cycle number (Ct) for each sample, compared against a standard curve generated from CpGenome Universal Methylated DNA (Chemicon International Inc, CA, USA). The percentage methylated of reference (PMR) value was calculated by dividing the *GENE/ACTB* ratio of a sample by the *GENE/ACTB* ratio of a positive control, CpGenome Universal Methylated DNA, and multiplying by 100. The methylation status of each sample was determined as positive when PMR>4 [29].

### Pyrosequencing quantitative methylation analysis

Pyrosequencing was applied to validate and dissect the methylation alterations in the observed target CpG regions of *p15^INK4b^* according to the manufacturer's instructions. Briefly, a biotin-labeled primer and bisulfite converted DNA were mixed and performed with PCR, allowing for isolation of the amplicon. Subsequently, the PCR products were denaturated and released to single strand products for pyrosequencing using the PyroMark Q24 system (Qiagen, Hilden, German). DNA methylation percentage at each CpG site was analyzed by PyroMark Q24 version 1.0.10 software in the CpG analysis mode. The *p15^INK4b^* forward primer: GGG AGG GTA ATG AAG TTG AGT; reverse primer: Biotin-CTA CCC CCC CCA CTA AAC ATA CCC TTA T; sequencing primers: TTG AGT TTA GGT TTT TTA GGA and GGA GTA GAG TGG GAA AGA A.

### RNA isolation and quantitative reverse-trasnscript polymerase chain reaction (RT-PCR)

Total RNA from whole blood cells was extracted using an RNeasy Mini Kit (Qiagen, Hilden, Germany) and 1 µg RNA was reverse transcribed with a PrimeScript RT reagent Kit (Takara Biotechnology, Tokyo, Japan). Relative quantification of gene expression was performed in duplicate. Specific primers for *p14^ARF^*, *p15^INK4b^*, *p16^INK4a^* and *GAPDH* were designed for relative quantitative RT-PCR (Table S2). The mRNA expression was determined using SYBR Premix Ex Taq™ (Takara Biotechnology, Tokyo, Japan). Ct values for each target gene were normalized to *GAPDH*. Given the low expression levels and the established splice variants of *ANRIL* transcripts, three specific primers and probes as previously designated were used (Table S2) [15]. As marked in Figure 1, these splices were proposed to indicate the expression of proximal, central and distal exons of *ANRIL*, since there is devoid of a deep understanding of *ANRIL* splice variants and an economic high-throughput approach to analyzing the full length of long non-coding RNA.

### Statistical analysis

The normal distribution of data was tested by Kolmogorov-Smirnov test. While the values of PMR and mRNA expression were highly skewed, Mann-Whitney U test was undertaken to examine differences between two groups. The significant differences between categorical variables were determined using χ^2^ test. Correlations between *p14^ARF^*, *p15^INK4b^*, *p16^INK4a^* and *ANRIL* expression were tested using the Spearman's nonparametric correlation test. ANOVA test and Bonferroni correction were then used to compare gene expression and PMR values across the genotypes. Logistic regression analysis including environmental and genetic risk factors (ie., age, gender, smoking, hypertension, diabetes, lipid profiles, genotype, candidate gene expression and methylation) was performed to identify the independent determinants of CAD. All statistics were performed with SPSS 14.0 (SPSS Inc, Chicago, IL, USA). A value of p<0.05 was considered significant (two tailed).

## Results

### Methylation of candidate genes and expression of *INK4/ARF* and *ANRIL* in patients with CAD

**Figure 2:**
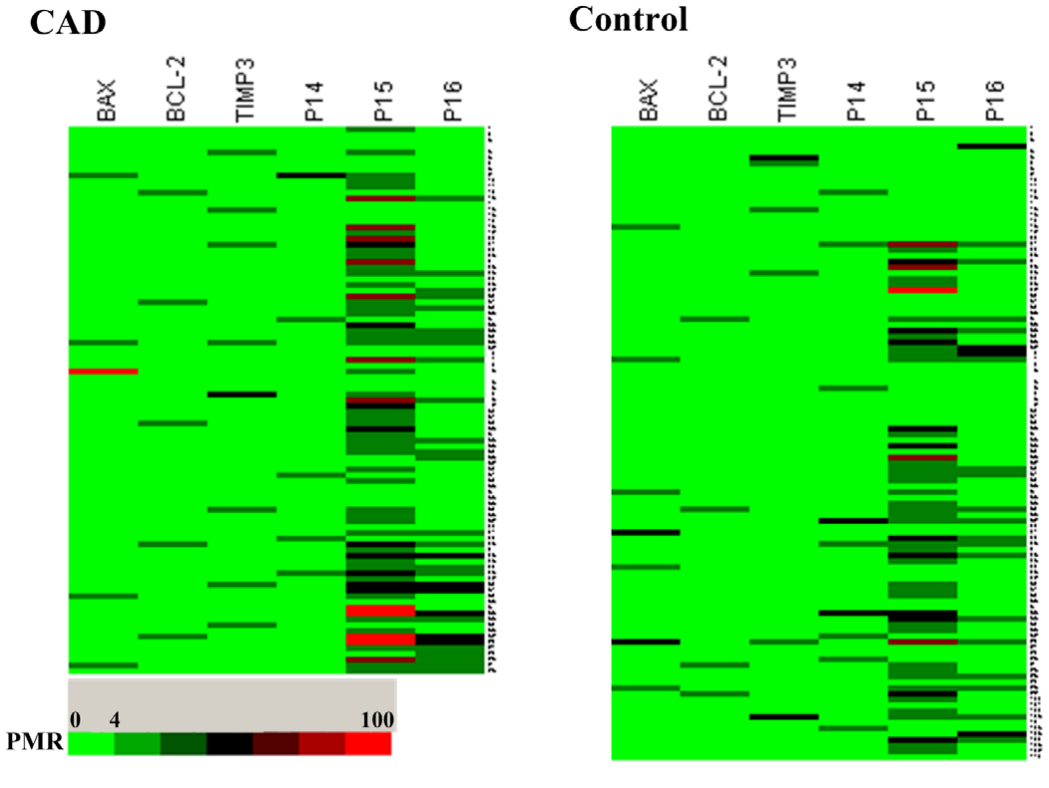
Heatmap for the CAD patients and controls. Six candidate loci are termed at the top. Details of MethyLight assay and PMR definition are delineated in the Methods section. The colored squares indicate the same range in PMR values as defined in the colorbar. DNA methylation levels are indicated by a color gradient, with the highest DNA methylation levels for each locus indicated in red and the lowest in deep green. The unmethylated loci (PMR<4) are indicated in green.

Baseline characteristics of the patients and controls are listed in Table 1. Their mean age was 64 years, 56.1% were male, 31.7% had a history of diabetes and 66.8% had a diagnosis of hyptertension. Apart from statin treatment in a pre-hospital setting (p<0.001), no significant differences in baseline characteristics were seen between the CAD patients and controls. Similarly, there was no discrepancy in the peripheral total white blood cell count, differential count and red blood cell profile between two groups (Table S3). First of all, for the purpose of identification of candidate hypermethylated genes in peripheral blood cells of CAD patients, we searched previous studies and explored whether these genes were hypermethylated in CAD patients. Seven predefined apoptosis-related genes (*LOX-1*, *CASP3*, *BCL-2*, *BAX*, *TIMP3*, *ANXA5* and *cIAP-3*), which were found hypermethylated *in vitro* [30], and *INK4/ARF* were screened for CpG island methylation events in a cohort of 40 CAD patients using methylation-specific PCR. While four genes were absences of methylation (*LOX-1*, *CASP3*, *ANXA5* and *cIAP-3*, data not shown), we then applied quantitative analyses to determine the methylation levels of the remaining three genes and *INK4/ARF*. As delineated in Figure 2, the methylation levels of *BCL-2*, *BAX*, *TIMP3* and *p14^ARF^* were at barely detectable levels in both CAD patients and controls. *p15^INK4b^* was the only one showing significant methylation among candidate genes in CAD patients.

**Table 1:** Clinical characteristics of patients with and without coronary artery disease.

	CAD (n = 95)	Control (n = 110)	p-value
Age, yrs	65.1±10.0	63.8±12.9	0.101
Male	56 (58.9)	59 (53.6)	0.445
BMI, kg/m^2^	24.5±3.4	24.3±3.2	0.755
Smoking	24 (25.3)	20 (18.2)	0.218
Diabetes	30 (31.6)	35 (31.8)	0.971
Fasting glucose, mmol/L	5.8±2.0	5.7±1.5	0.465
Hypertension	64 (67.4)	73 (66.4)	0.879
Triglyceride, mmol/L	1.7±0.9	1.6±0.8	0.977
Cholesterol, mmol/L	4.4±1.0	4.7±1.0	0.737
LDL-C, mmol/L	2.4±0.9	2.7±0.8	0.339
HDL-C, mmol/L	1.1±0.3	1.2±0.3	0.683
BUN, mmol/L	5.9±2.4	6.2±3.0	0.482
Creatinine, umol/L	88.3±42.8	78.6±27.0	0.062
Statin	29 (30.5)	7 (6.4)	**<0.001**
ACEI/ARB	41 (43.2)	38 (34.5)	0.206

Values are mean ± SD or n (%).

Abbreviations: ACEI = angiotensin-converting enzyme inhibitors; ARB = angiotensin II receptor blocker; BMI = body mass index; BUN = blood urine nitrogen; CAD = coronary artery disease; HDL-C = high-density lipoprotein cholesterol; LDL-C = low-density lipoprotein cholesterol.

**Figure 3:**
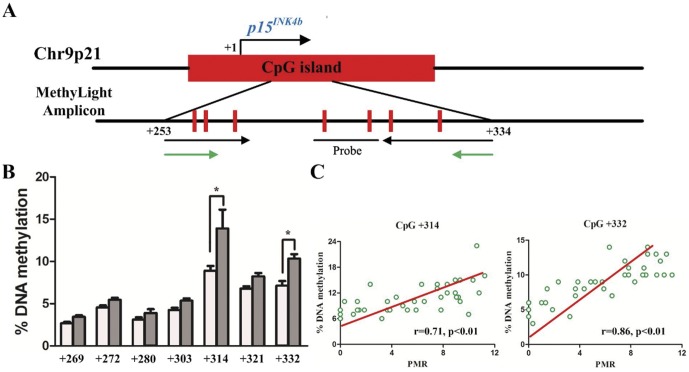
Pyrosequencing to identify the methylated CpG sites of *p15^INK4b^*. A. Schematic diagram of the CpG island within *p15^INK4b^*. Seven CpG sites detected by pyrosequencing are indicated in red. Black and green arrows indicate the PCR primer pairs of MethyLight and pyrosequencing respectively. B. DNA methylation levels at each CpG site in percentage are analyzed by pyrosequencing in 22 randomly selected controls (white bar) and 22 CAD patients (grey bar). Date are presented as mean ± SD. * p<0.05 vs. controls. C. Correlation of *p15^INK4b^* methylation levels measured by MethyLight and pyrosequencing at CpG +314 and +332. Correlation efficient (r) and p value as indicated.

Next, the gene expression and methylation on Chr9p21 were compared between CAD patients and controls (Table 2). A dramatically increase in methylation levels of *p15^INK4b^* in the CAD group was observed compared with control (p = 0.006). CAD patients had a higher expression level of *p14^ARF^* (p = 0.048), yet no differences in *p16^INK4a^* methylation and expression of other genes were observed. Multivariate regression analysis showed that rs10757274 (OR = 1.80; 95% CI: 1.21–3.00), *p15^INK4b^* methylation (OR = 2.55; 95% CI: 1.26–5.01) and statin were the independent determinants of CAD (Table 3). For the purpose of validation and dissection of *p15^INK4b^* methylation percentages at interested CpG sites, we then applied pyrosequencing in 22 randomly selected controls and 22 CAD patients. DNA methylation percentages were obtained for seven CpGs covering 81 bp of *p15^INK4b^* (Figure 3A). As shown in Figure 3B, five analyzed CpG sites, located 269, 272, 280, 303 and 321 bp downstream (CpG +269, +272, +280, +303 and +321) of the transcription start site, were slightly methylated in both groups without between-group difference, whereas the degrees of methylation at CpG +314 and +332 were significantly increased in CAD patients compared with controls (p = 0.01 and 0.03, respectively). Additionally, the pyrosequencing results at CpG +314 and +332 were tightly correlated with *p15^INK4b^* methylation levels measured by MethyLight assay (Figure 3C).

**Table 2:** Methylation status of *p15^INK4b^* and *p16^INK4a^* and candidate gene expression in controls and CAD patients.

	CAD (n = 95)	Controls (n = 110)	p-value
PMR values			
*p15^INK4b^*	5.93 [2.21–9.47]	3.11 [0–8.08]	**0.006**
*p16^INK4a^*	0 [0–7.86]	0	0.075
Gene expression			
*p14^ARF^*	0.21 [0.05–0.87]	0.10 [0.04–0.37]	**0.048**
*p15^INK4b^*	0.37 [0.12–1.03]	0.49 [0.10–1.57]	0.252
*p16^INK4a^*	0.58 [0.15–6.38]	2.00 [0.19–9.67]	0.146
*ANRIL exon 1–5*	0.38 [0.11–1.52]	0.30 [0.06–1.09]	0.312
*ANRIL exon 4–5*	0.07 [0.01–0.30]	0.06 [0.01–0.26]	0.711
*ANRIL exon 17–18*	0.04 [0.03–0.09]	0.06 [0.03–0.19]	0.605

Continuous data are expressed as median and interquartile range.

Abbreviations: ANRIL = antisense non-coding RNA in the INK4 locus; PMR = percentage methylated of reference.

**Table 3:** Multivariate regression analysis of independent determinants of CAD.

	OR	95% CI	p-value
Age (>65 yrs)	1.37	0.72–2.67	0.354
Male	0.88	0.47–1.76	0.632
BMI (>25 kg/m^2^)	1.01	0.50–2.11	0.938
Smoking	1.40	0.60–3.19	0.408
Hypertension	1.03	0.49–2.13	0.917
Diabetes	0.70	0.36–1.42	0.342
Triglyceride (>1.7 mmol/L)	1.33	0.77–2.57	0.521
Cholesterol (>5.7 mmol/L)	0.33	0.68–1.17	0.079
LDL-C (>3.64 mmol/L)	4.88	0.82–32.49	0.108
HDL-C (<0.91 mmol/L)	0.52	0.28–1.26	0.162
BUN	1.49	0.47–3.06	0.316
Creatinine	0.82	0.48–1.40	0.521
Statin	5.63	2.02–14.87	**0.001**
ACEI/ARB	1.20	0.60–3.09	0.530
rs10757274	1.80	1.21–3.00	**0.009**
*p15^INK4b^* M*	2.55	1.26–5.01	**0.015**
*p16^INK4a^* M*	1.14	0.59–2.36	0.819
*ANRIL exon 1–5*	0.81	0.40–1.70	0.736
*ANRIL exon 4–5*	0.80	0.41–1.75	0.719
*ANRIL exon 17–18*	0.83	0.43–1.57	0.780

* *p15^INK4b^*/*p16^INK4a^* M means *p15^INK4b^*/*p16^INK4a^* methylation defined as PMR>4.

Abbreviations as in Table 1 and 2.

**Figure 4:**
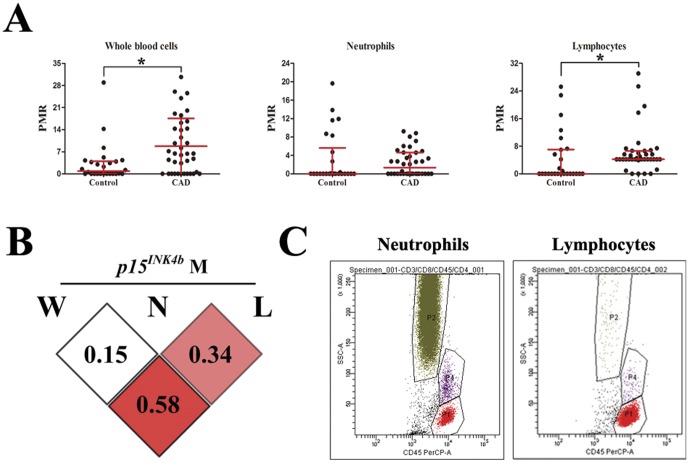
*p15^INK4b^* methylation in whole blood cells, neutrophils and lymphocytes. A. Comparison of *p15^INK4b^* methylation between controls and CAD patients in whole blood cells, neutrophils and lymphocytes. B. Correlation of *p15^INK4b^* methylation among whole blood cells, neutrophils and lymphocytes. Red regions indicate positive significant correlation while no significant correlation is shown as white. C. Representative images of purified neutrophils at the left and lymphocytes at the right by fluorescence-activated cell sorting. Neutrophils are shown as brown green dots, monocytes as purple dots and lymphocytes as red dots. *p<0.05 vs. controls. Abbreviation: L = lymphocytes; M = methylation; N = neutrophils; W = whole blood cells.

To determine which types of leukocytes contributed to *p15^INK4b^* methylation in peripheral blood cells, genomic DNA was extracted from whole blood cells, purified neutrophils and lymphocytes. Neutrophils and lymphocytes from peripheral blood were >93% pure by fluorescence-activated cell sorting and morphology (Figure 4C). Baseline characteristics of the second sample are listed in Table S4. As shown in Figure 4A, a significant increase in *p15^INK4b^* methylation was observed in both whole blood cells and lymphocytes (p = 0.011 and 0.013, respectively). Likewise, *p15^INK4b^* methylation in lymphocytes was highly correlated with that in whole blood cells (r = 0.54, p = 0.002) (Figure 4B).

We further investigated the association between the number of culprit vessels and *ANRIL* expression and *p15^INK4b^/p16^INK4a^* methylation. Neither *p15^INK4b^* nor *p16^INK4a^* had statistically significant difference in methylation among the severity of CAD, whereas there was a gradual increase in *ANRIL exon 4–5* expression as number of culprit vessels increased (p = 0.042) (Figure S1).

### Effect of risk genotype on *p15^INK4b^/p16^INK4a^* methylation and *ANRIL* expression

Further, we assessed the association between CAD risk genotypes on Chr9p21 and methylation of *p15^INK4b^/p16^INK4a^*, and expression of *INK4/ARF* and *ANRIL* (Table 4). The distribution of genotypes in patients with CAD and controls was in Hardy-Weinberg equilibrium (p = 0.943). Consistent with previous data [2], [12], rs10757274 GG risk genotype was significantly associated with CAD (p = 0.003). Compared with carriers of AA and AG genotypes, GG genotype carriers (0.79 [0.28–2.33]) had markedly elevated levels of *ANRIL exon 1–5* expression (GG: 0.79 [0.28–2.33], AG: 0.29 [0.08–0.97], AA: 0.15 [0.02–0.85] respectively) (p = 0.009) (Table 4). In contrast, there were no differences in other gene expression and DNA methylation among three genotypes of rs10757274 (Table 4).

**Table 4:** Association of rs10757274 on chromosome 9p21 with CAD cases and controls.

	rs10757274			
	AA (n = 50)	AG (n = 103)	GG (n = 52)	p-value
CAD/Controls, n	15/35	47/56	33/19	**0.003**
PMR values				
*p15^INK4b^*	4.03 [0–8.65]	4.07 [0–8.34]	6.12 [0–9.69]	0.257
*p16^INK4a^*	0	0	0 [0–9.80]	0.072
Gene expression				
*p14^ARF^*	0.08 [0.02–0.37]	0.13 [0.04–0.79]	0.14 [0.05–0.75]	0.225
*p15^INK4b^*	0.41 [0.09–1.48]	0.42 [0.04–0.79]	0.39 [0.06–0.94]	0.621
*p16^INK4a^*	1.78 [0.13–10.40]	0.94 [0.19–8.11]	1.29 [0.14–0.75]	0.722
*ANRIL exon 1–5*	0.15 [0.02–0.85]	0.29 [0.08–0.97]	0.79 [0.28–2.33]	**0.009** *
*ANRIL exon 4–5*	0.06 [0–0.20]	0.07 [0.01–0.33]	0.05 [0–0.21]	0.315
*ANRIL exon 17–18*	0.04 [0.01–0.14]	0.06 [0.04–0.18]	0.04 [0.03–0.06]	0.408

* The difference in *ANRIL exon 1–5* expression between carriers of AG and GG genotypes holds after a Bonferroni correction (p = 0.007).

Continuous data are expressed as median and interquartile range.

Abbreviations as in Table 2.

### Correlation among *p15^INK4b^/p16^INK4a^* methylation and gene expression on Chr9p21

**Figure 5:**
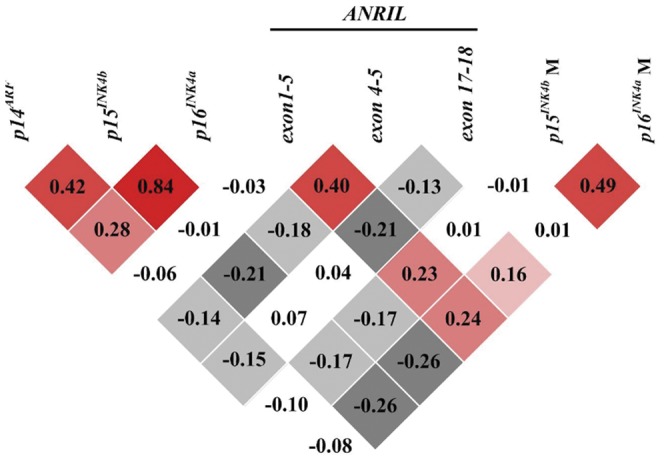
Correlation among *p15^INK4b^*/*p16^INK4a^* methylation and gene expression on chromosome 9p21. Red regions indicate positive significant correlation and grey regions indicate inverse significant correlation, while no significant correlation is shown as white.

There was a strong correlation among *INK4/ARF* expression (r = 0.28 to 0.84, p<0.001) (Figure 5). *p15^INK4b^* and *p16^INK4a^* methylation was inversely correlated with their corresponding genes expression (r = −0.17, p = 0.011 and r = −0.26, p = 0.002, respectively), which was in accordance with the theory that aberrant methylation of the CpG islands at promoters and exons is linked to loss of genes expression and their function. The significant associations of *p15^INK4b^/p16^INK4a^* methylation with serum levels of lipid profile or glucose were not found (data not shown).

### Association between *ANRIL* expression and *p15^INK4b^/p16^INK4a^* methylation

**Figure 6:**
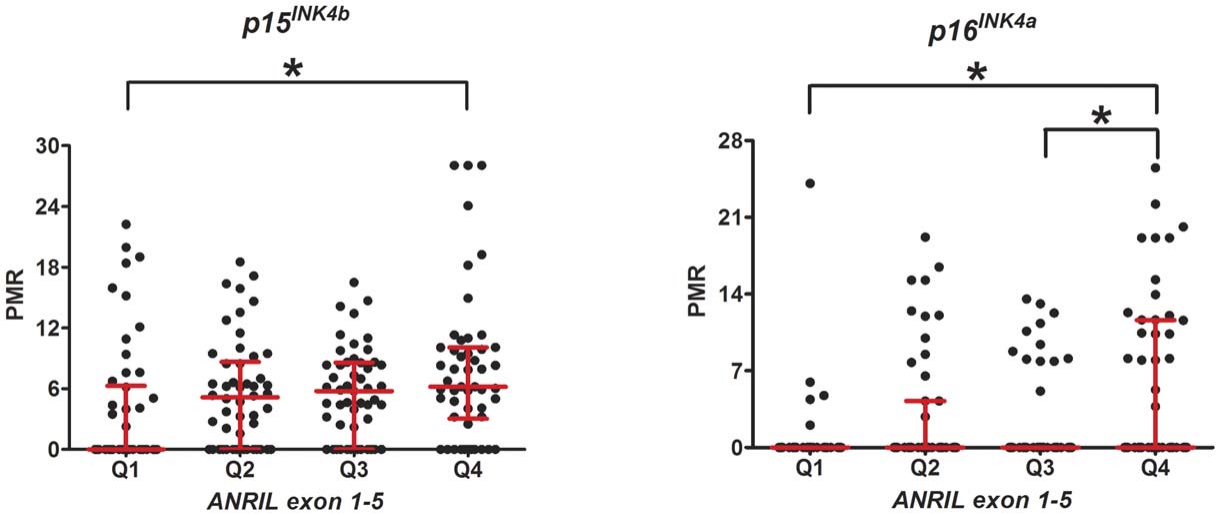
Effect of *ANRIL exon 1–5* on *p15^INK4b^* and *p16^INK4a^* methylation. The PMR values of *p15^INK4b^* and *p16^INK4a^* methylation are compared among categories created by quartiles of *ANRIL exon 1–5* distribution (Q1–Q4). Red lines indicate median and interquartile range. *p<0.01. Note that p values are significance after correction for multiple comparisons by Bonferroni analysis.

Another intriguing observation in our study was the positive significant correlation between *ANRIL exon 1–5* expression and *p15^INK4b^* and *p16^INK4a^* methylation (r = 0.23, p = 0.001 and r = 0.24, p = 0.001, respectively). Similarly, *ANRIL exon 4–5* expression was tightly associated with *p16^INK4a^* methylation and *p15^INK4b^/p16^INK4a^* mRNA expression but not *p15^INK4b^* methylation (Figure 5). When the whole enrolled cases including CAD patients and controls were regrouped according to their quartiles of *ANRIL exon 1–5* distribution, there was a slight increase in *p15^INK4b^* methylation in subjects in the upper quartile of *ANRIL exon 1–5* expression (p = 0.009). Likewise, we observed a stepwise increase in the levels of *p16^INK4a^* methylation as *ANRIL exon 1–5* expression elevated (p<0.001) (Figure 6).

## Discussion

This work presents the first quantitative analysis of specific genes methylation in CAD. Our data indicated that *p15^INK4b^* methylation was an important event in atherosclerosis, and such potential bridge between genotype and *p15^INK4b^* methylation might be mediated by altered expression of *ANRIL*.

Although epigenetic changes are of crucial importance in the pathophysiology of atherosclerosis in response to multiple genetic and modifiable risk factors, there is still little data about the methylation status at specific loci [31]. Studies attempting to explore global methylation in patients with cardiovascular diseases did not reach a consensus, which mainly resulted from variance in subjects' selection criteria of each study [32], [33], [34], [35]. In the present study, CAD patients diagnosed by angiography and age-, sex- and concomitant diseases-matched participants were enrolled, thereby avoiding the confounding effects of established risk factors on DNA methylation. Additionally, the results from previous studies failed to identify whether the methylation events emerged at specific loci. A previous model suggested that the exposure of HUVECs to ox-LDL induced other candidate gene methylation, yet these results could not be replicated in CAD patients so far [30].

*INK4/ARF* transcripts participate in the regulation of cell cycle arrest via p53 and Rb pathways and play important roles in cell proliferation and senescence [36]. In our study, *p15^INK4b^*, whose hypermethylation has been proved to involve in the initiation and development of multiple types of cancers, was also strikingly hypermethylated in CAD patients compared with controls. Because there is no discrepancy observed in cellular composition of the blood samples between two groups, it is thus plausible that the changes in *p15^INK4b^* methylation and gene expression depend on alterations in one or multiple given blood cell types. Therefore, another central conundrum is which types of leukocytes in peripheral blood are responsible for *p15^INK4b^* methylation since epigenomic alterations vary from cell type to cell type in contrast to genetic variants. Pointing to this question, our results showed highly parallel levels of *p15^INK4b^* methylation observed in whole blood cells and lymphocytes after careful collections of neutrophils and lymphocytes from peripheral blood. Among the differential leukocytes, lymphocytes have been considered to mediate immune and inflammatory response through altered DNA methyltransferases expression and alterations to inflammation-related DNA methylation [37], [38]. Furthermore, the observations that significantly increased *p15^INK4b^* methylation in CAD patients coincided with decreased *p15^INK4b^* and *p16^INK4a^* expression could be predominantly explained by the fact that *p15^INK4b^* methylation could repress *INK4/ARF* expression, subsequently contributing to atherosclerosis. Prior studies conducted in *INK4/ARF* knockout mice model also found that either deficiency of *p14^ARF^* or *p16^INK4a^* was closely associated with atherosclerosis, thus providing an elegant rationale for our preliminary results [39], [40]. Nevertheless, the *p15^INK4b^* and *p16^INK4a^* expression failed to be inversely correlated with CAD. Indeed, notwithstanding a significant increase of *p15^INK4b^* methylation in CAD patients compared to that in controls, the average methylation level in CAD patients modestly exceeded the normal value. The significant but rather small changes in *p15^INK4b^* methylation were subsequently dissected by pyrosequencing, finding that two of the seven observed CpGs (CpG +314 and +332) were markedly hypermethylated in CAD patients compared with controls. Indeed, the identification of CpG +314 and +332 hypermethylation at *p15^INK4b^* is of important value, whereas two sites seem incapable of remarkably attenuating *p15^INK4b^* expression in CAD patients compared with controls.

All available evidence to date have indicated that long non-coding RNAs attenuated expression of associated genes through diverse mechanisms such as heterochromatin formation, histone modifications, RNA interference, DNA methylation, etc. [19], [20], [41], [42], [43]. Our findings presented herein suggested that specific *ANRIL* species had close associations with *p15^INK4b^* and *p16^INK4a^* methylation, giving rise to the down-regulation of the corresponding gene expression. It is demonstrated that polycomb repressor complexes (PRCs) are of particular importance in repressing *INK4/ARF* expression. Current *in vivo* and *in vitro* studies unveiled that known members of PRC family, such as EZH2, CBX7 and SUZ12, contributed to initiation and maintenance of DNA hypermethylation via interaction with DNA methyltransferases [44], [45], [46]. On the other side of the coin, *ANRIL* may directly recruit some components of PRC family, ensuing repression of *p15^INK4b^* and *p16^INK4a^* through histone modifications [19], [20], which are associated with aberrant methylation [47]. It is thus conceivable that *ANRIL*, coupled with PRCs, may cause a decrease in expression of *p15^INK4b^* and *p16^INK4a^* by programming epigenome including DNA methylation and histone modifications.

Together with the knowledge of CAD-associated SNPs adjacent to *INK4/ARF* locus, we came to the hypothesis that individuals may have an intrinsic propensity to *p15^INK4b^/p16^INK4a^* methylation and risk SNPs located in Chr9p21 may function through the contribution of *ANRIL* to neighboring *INK4/ARF* methylation. In this regard, our results found *ANRIL exon 1–5* in carriers of risk genotype was overexpressed and had a strong association with *p15^INK4b^/p16^INK4a^* methylation. Although the mechanism underlying genetic variants remains ambiguous, previous studies have provided evidence that SNP genotype directly contributes to *ANRIL* expression but indirectly to *INK4/ARF* expression in Caucasian population [14], [15], [48]. In line with these observations, we found a marked association of rs10757274 genotype with *ANRIL* expression rather than *p15^INK4b^*/*p16^INK4a^* methylation and expression in Chinese population. Recently, an *in vitro* experiment showed that *ANRIL* knockdown suppressed *p15^INK4b^* and *p16^INK4a^* expression, which in turn inhibited VSMCs proliferation [11]. *In vivo*, Visel et al. created a mouse model with targeted deletion of the orthologous 70 kb non-coding interval on mouse chromosome 4 to investigate the causality between human non-coding risk interval linked to CAD susceptibility and the neighboring *INK4/ARF* expression [10]. A convincing conclusion of this work was that deletion of non-coding CAD risk interval could attenuate expression of *p15^INK4b^* and *p16^INK4a^* through a *cis*-acting effect, consequently triggering excessive proliferation of VSMCs. Morever, it will be of future interest to determine the potential mechanisms how *p15^INK4b^*, *p16^INK4a^* methylations were regulated by *ANRIL* and whether the causality, if any, between *p15^INK4b^*, *p16^INK4a^* methylation and *ANRIL* expression is bidirectional.

Our findings add to the body of knowledge on epigenetic changes in CAD, mainly *p15^INK4b^* methylation on Chr9p21. However, our study poses several limitations that deserved further consideration. First and above all, this study was retrospective in nature and increased the likelihood of selection bias, although the patients in two groups were matched on up to 15 variables to adjust for the differences in baseline data. Therefore, the *INK4/ARF* methylation and its influence on risk for CAD need to be investigated by more studies such as cohort studies.

## Conclusions

*p15^INK4b^* methylation is associated with CAD and *ANRIL* expression, both of which are directly affected by gene polymorphisms on Chr9p21. These results point to a potential role of epigenetic changes as mediators from Chr9p21 polymorphisms to CAD.

## Supporting Information

Figure S1 **Changes into** ***p15^INK4b^/p16^INK4a^*** **methylation and** ***ANRIL*** **expression according to the number of culprit vessels.** A and B. Association of *p15^INK4b^/p16^INK4a^* Methylation with the number of culprit vessels. The box plots display median and interquartile range and the minimum and maximum levels as horizontal lines outside the box. C and D. Association of *ANRIL exon 1–5 and 4–5* expression with the number of culprit vessels. The histograms indicate median and interquartile range.
(TIF)

Table S1 **Summary of MethyLight primer and probe sequences.** The CpG sites examined are highlighted in bold. * *ACTB*: *β-Actin*.
(DOC)

Table S2 **Summary of pimer and/or probe sequences used for quantitative RT-PCR.**
(DOC)

Table S3 **Comparison of cellular composition of the blood samples between CAD patients and controls.** The proportions of neutrophils, lymphocytes, monocytes, eosnophils and basophils in white differential count are documented in each participant. Data are presented as mean ± SD. Abbreviations: RBCs = red blood cells; WBCs = white blood cells.
(DOC)

Table S4 **Baseline characteristics of the second sample.** Values are mean ± SD or n (%).Abbreviations as in Table 1 and S3.
(DOC)
