# Supplementary figures and images for: Methylation of p15INK4b and Expression of ANRIL on Chromosome 9p21 Are Associated with Coronary Artery Disease
Source: PLoS One. 2012 Oct 16;7(10):e47193. doi: 10.1371/journal.pone.0047193 (PMC3473029; doi:10.1371/journal.pone.0047193)

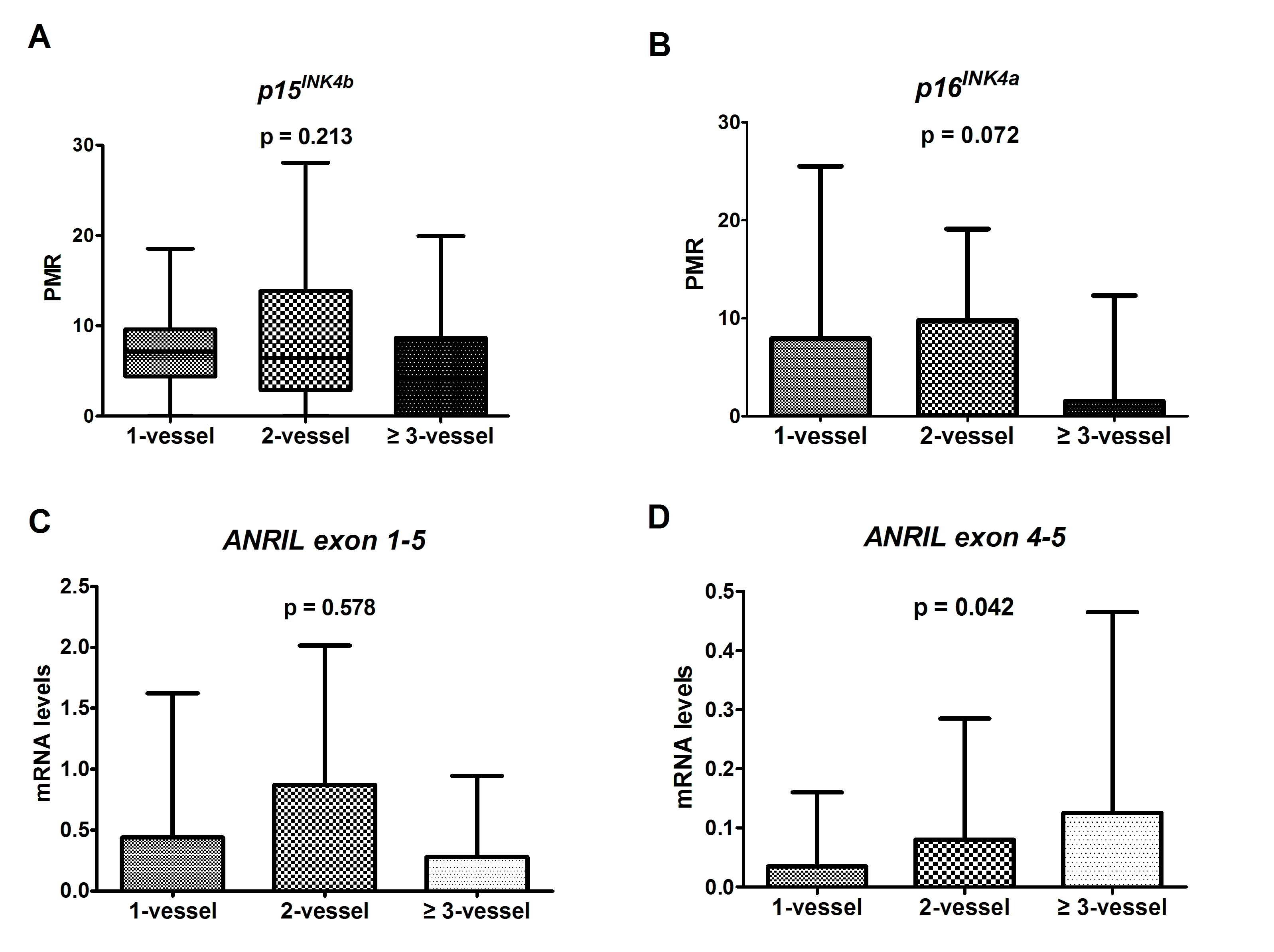

Supplement: Figure S1 — Changes into p15INK4b/p16INK4a methylation and ANRIL expression according to the number of culprit vessels. A and B. Association of p15INK4b/p16INK4a Methylation with the number of culprit vessels. The box plots display median and interquartile range and the minimum and maximum levels as horizontal lines outside the box. C and D. Association of ANRIL exon 1–5 and 4–5 expression with the number of culprit vessels. The histograms indicate median and interquartile range. (TIF) [file pone.0047193.s001.tif]
