# Supplementary material for: Methylation of p15INK4b and Expression of ANRIL on Chromosome 9p21 Are Associated with Coronary Artery Disease
Source: PLoS One. 2012 Oct 16;7(10):e47193. doi: 10.1371/journal.pone.0047193 (PMC3473029; doi:10.1371/journal.pone.0047193)
Supplement: Table S1 — Summary of MethyLight primer and probe sequences. The CpG sites examined are highlighted in bold. * ACTB: β-Actin. (DOC) [file pone.0047193.s002.doc]

**Table S1. Summary of MethyLight primer and probe sequences**

| Gene (Ref.) | GenBank accession number | Primer (5’→3’) | Probe |
| --- | --- | --- | --- |
| *BAX* (1) | NM_138762 | Forward: **CG**GGAGGTAGA**CG**GG**CG**  Reverse: CAACCCCAAAC**CG**ATAAAAAAA | 6FAM-AGGG**CG**AGTTTTTT**CG**TCGGTT**CG**-BHQ1 |
| *BCL-2* (1) | NM_000633 | Forward: T**CG**TATTT**CG**GGATTCGGTC  Reverse: AACTAAA**CG**CAAACCC**CG**C | 6FAM-A**CG**A**CG**C**CG**AAAACAAC**CG**AAATCTACA-TAMRA |
| *TIMP3* (2) | U33110 | Forward: G**CG**T**CG**GAGGTTAAGGTTGTT  Reverse: CTCTCCAAAATTAC**CG**TA**CGCG** | 6FAM-AACT**CG**CT**CGC**C**CG**C**CG**AA-TAMRA |
| *p14^ARF^* (1) | AF082338 | Forward: A**CG**GG**CG**TTTT**CG**GTAGTT  Reverse: C**CG**AACCTCCAAAATCT**CG**A | 6FAM-**CG**ACTCTAAACCCTA**CG**CA**CGCG**AAA-BHQ1 |
| *p15^INK4b^* (2) | S75756 | Forward: AGGAAGGAGAGAGTG**CG**T**CG**  Reverse: **CG**AATAATCCAC**CG**TTAAC**CG** | 6FAM-TTAA**CG**ACACTCTTCCCTTCTTTCCCA**CG**-TAMRA |
| *p16^INK4a^* (2) | NM_000077 | Forward: TGGAATTTT**CG**GTTGATTGGTT  Reverse: AACAA**CG**TCC**GC**ACCTCCT | 6FAM-ACC**CG**ACCC**CG**AAC**CGCG**-TAMRA |
| *ACTB* ^*^ (1) | Y00474 | Forward: TGGTGATGGAGGAGGTTTAGTAAGT  Reverse: AACCAATAAAACCTACTCCTCCCTTAA | 6FAM-ACCACCACCCAACACACAATAACAAACACA-BHQ1 |

The CpG sites examined are highlighted in bold.

* *ACTB*: *β-Actin*.

1. Friedrich MG, Weisenberger DJ, Cheng JC, et al. Detection of methylated apoptosis-associated genes in urine sediments of bladder cancer patients. Clin Cancer Res 2004;10:7457-65.

2. Eads CA, Lord RV, Wickramasinghe K, et al. Epigenetic patterns in the progression of esophageal adenocarcinoma. Cancer Res 2001;61:3410-8.
