# Supplementary material for: Methylation of p15INK4b and Expression of ANRIL on Chromosome 9p21 Are Associated with Coronary Artery Disease
Source: PLoS One. 2012 Oct 16;7(10):e47193. doi: 10.1371/journal.pone.0047193 (PMC3473029; doi:10.1371/journal.pone.0047193)
Supplement: Table S2 — Summary of pimer and/or probe sequences used for quantitative RT-PCR. (DOC) [file pone.0047193.s003.doc]

**Table S2. Summary of pimer and/or probe sequences used for q**uantitative RT-PCR

| Gene (Ref.) | Primer and/or probe sequence (5’→3’) |
| --- | --- |
| *p14^ARF^*  ENST00000530628 | Forward: GCCAGGGGCGCCCGCCGCTG  Reverse: GGCCCGGTGCAGCACCACCA |
| *p15^INK4b^*  ENST00000276925 | Forward: AGTCAACCGTTTCGGGAGGC  Reverse: ACCACCAGCGTGTCCAGGAAG |
| *p16^INK4a^*  ENST00000304494 | Forward: GCTGCCCAACGCACCGAATA  Reverse: ACCACCAGCGTGTCCAGGAA |
| *GADPH* | Forward: ACGGATTTGGTCGTATTGGG  Reverse: TGATTTTGGAGGTATCTCGC |
| *ANRIL exon 17-18*  *NR_003529* (1) | Forward: CAGAGCAATTCCAGTGCAAG  Reverse: GATTTGCAAAAACAGCTG  Probe: 6FAM-CTGCTACATGGAGGCTAGGGCCAGAGTCA-TAMRA |
| *ANRIL exon 4-5*  *DQ485454* (1) | Forward: TGTACTTAACCACTGGACTACCTGCC  Reverse: CTTTGATCTCTGCTGTTGAATCAGAATG  Probe: 6FAM-TGCCCTGTCGAGGAACAGCTAAGTGTCCCT-TAMRA |
| *ANRIL exon 1-5*  *EU741058* (1) | Forward: TGCCGGAGCTGTCGACCC  Reverse: CTTTGATCTCTGCTGTTGAATCAGAATG  Probe: 6FAM-CGGCCTGGCGCCGGACTAGTGTC-TAMRA |
| *β-Actin* (1)  ENST00000331789 | Forward: CCTGGCACCCAGCACAAT  Reverse: GCCGATCCACACGGAGTACTT  Probe: 6FAM-ATCAAGATCATTGCTCCTCCTGAGCGCA-TAMRA |

1. Holdt LM, Beutner F, Scholz M, et al. ANRIL expression is associated with atherosclerosis risk at chromosome 9p21. Arterioscler Thromb Vasc Biol 2010;30:620-7.
