# Supplementary material for: Methylation of p15INK4b and Expression of ANRIL on Chromosome 9p21 Are Associated with Coronary Artery Disease
Source: PLoS One. 2012 Oct 16;7(10):e47193. doi: 10.1371/journal.pone.0047193 (PMC3473029; doi:10.1371/journal.pone.0047193)
Supplement: Table S3 — Comparison of cellular composition of the blood samples between CAD patients and controls. The proportions of neutrophils, lymphocytes, monocytes, eosnophils and basophils in white differential count are documented in each participant. Data are presented as mean ± SD. Abbreviations: RBCs = red blood cells; WBCs = white blood cells. (DOC) [file pone.0047193.s004.doc]

**Table S3. Comparison of cellular composition of the blood samples between CAD patients and c**ontrols

|  | CAD (n=95) | Control (n=110) | p-value |
| --- | --- | --- | --- |
| RBCs, *10^12^/L | 4.08 ± 0.56 | 4.27 ± 0.50 | 0.48 |
| WBCs, *10^9^/L | 6.49 ± 2.40 | 7.09 ± 1.75 | 0.18 |
| Neutrophils, % | 60.88 ± 8.26 | 60.60 ± 10.72 | 0.91 |
| Lymphocytes, % | 28.22 ± 8.86 | 29.78 ± 9.91 | 0.21 |
| Monocytes, % | 8.31 ± 2.35 | 7.78 ± 1.56 | 0.18 |
| Eosnophils, % | 2.49 ± 1.60 | 2.36 ± 1.58 | 0.86 |
| Basophils, % | 0.22 ± 0.16 | 0.24 ± 0.18 | 0.54 |

The proportions of neutrophils, lymphocytes, monocytes, eosnophils and basophils in white differential count are documented in each participant. Data are presented as mean ± SD.

Abbreviations: RBCs = red blood cells; WBCs = white blood cells.
