# Supplementary material for: Methylation of p15INK4b and Expression of ANRIL on Chromosome 9p21 Are Associated with Coronary Artery Disease
Source: PLoS One. 2012 Oct 16;7(10):e47193. doi: 10.1371/journal.pone.0047193 (PMC3473029; doi:10.1371/journal.pone.0047193)
Supplement: Table S4 — Baseline characteristics of the second sample. Values are mean ± SD or n (%).Abbreviations as in Table 1 and S3. (DOC) [file pone.0047193.s005.doc]

**Table S4. Baseline characteristics of the second sample**

|  | CAD (n=38) | Control (n=26) | p-value |
| --- | --- | --- | --- |
| Age, yrs | 63.8± 10.1 | 63.8 ± 8.0 | 0.998 |
| Male | 25 (65.8) | 12 (46.2) | 0.118 |
| BMI, kg/m^2^ | 24.7 ± 4.2 | 24.4 ± 4.7 | 0.788 |
| Smoking | 15 (39.5) | 5 (19.2) | 0.086 |
| Diabetes | 20 (52.6) | 10 (38.5) | 0.265 |
| Fasting glucose, mmol/L | 5.9 ± 2.0 | 5.1 ± 1.5 | 0.465 |
| Hypertension | 32 (84.2) | 16 (61.5) | **0.040** |
| Triglyceride, mmol/L | 2.0 ± 1.9 | 1.4 ± 0.5 | 0.183 |
| Cholesterol, mmol/L | 4.3 ± 1.2 | 4.6 ± 1.1 | 0.299 |
| LDL-C, mmol/L | 2.4 ± 0.9 | 2.7 ± 0.8 | 0.105 |
| HDL-C, mmol/L | 1.0 ± 0.3 | 1.1 ± 0.3 | 0.305 |
| BUN, mmol/L | 5.4 ± 2.3 | 5.3 ± 1.5 | 0.856 |
| Creatinine, umol/L | 76.1 ± 25.7 | 70.5 ± 21.9 | 0.420 |
| Statin | 12 (31.6) | 2 (7.7) | **0.023** |
| RBCs, *10^12^/L | 4.28 ± 0.62 | 4.36 ± 0.53 | 0.470 |
| WBCs, *10^9^/L | 6.91 ± 2.00 | 7.22 ± 3.00 | 0.629 |
| Neutrophils, % | 64.22 ± 10.93 | 62.09 ± 8.35 | 0.432 |
| Lymphocytes, % | 25.00 ± 8.21 | 28.32 ± 8.78 | 0.149 |
| Monocytes, % | 7.17 ± 2.21 | 6.75 ± 2.10 | 0.470 |
| Eosnophils, % | 2.01 ± 1.52 | 1.79 ± 1.08 | 0.551 |
| Basophils, % | 0.24 ± 0.17 | 0.30 ± 0.18 | 0.215 |

Values are mean ± SD or n (%).

Abbreviations as in Table 1 and S3.
